# Tinnitus and cell phones: the role of electromagnetic radiofrequency radiation^☆^

**DOI:** 10.1016/j.bjorl.2015.11.014

**Published:** 2016-02-09

**Authors:** Seyed Mohammad Javad Mortazavi, Seyed Ali Reza Mortazavi

**Affiliations:** a Ionizing and Non-ionizing Radiation Protection Research Center (INIRPRC), Shiraz University of Medical Sciences, Shiraz, Iran; b Student Research Committee, School of Medicine, Shiraz University of Medical Sciences, Shiraz, Iran

Dear Editor:

We read with great interest an article by Medeiros and Sanchez entitled “Tinnitus and cell phones: the role of electromagnetic radiofrequency radiation”, published as an “article in press” in the Brazilian Journal of Otorhinolaryngology (http://dx.doi.org/10.1016/j.bjorl.2015.04.013). The authors of this well-structured challenging paper have reviewed the role of electromagnetic radiofrequency (RF) radiation emitted by cell phones on the occurrence of Tinnitus. Based on the evidence presented by authors, they suggested that to prevent auditory damage and the onset or worsening of tinnitus, mobile phones should be used with caution. Although the paper published by Medeiros and Sanchez can be recognized as a remarkable contribution in the field of otorhinolaryngology, it has some shortcomings. First of all, the authors have addressed the issue of the so called adaptive response “*Any proven harmful effect can have wide-ranging implications, due to the universal exposure to EMRFR, …, In contrast, a study published in 1992 showed substantial evidence that when pre-exposed to low doses of DNA-damaging factors such as ionizing radiation, ultraviolet light, alkylating agents, and oxidants, cells can develop an adaptive response, with consequently greater resistance to higher doses of aggressive agents*”. Over the past several years, our lab has performed experiments on the health effects of exposure of animal models and humans to different sources of electromagnetic fields such as cellular phones, mobile base stations, mobile phone jammers, laptop computers, radars, dentistry cavitrons and MRI. We have previously reported that when living organisms pre-exposed to either low doses of ionizing radiation^1^, ^2^ or low levels of non-ionizing radiation,^3^ receive a relatively high dose later, the negative biological effects will be less than if they were exposed to the large dose alone. Therefore, when there is no challenge dose (lack of exposure to a high dose after receiving a low dose), adaptive response cannot be observed. Based on these points, instead of using “adaptive response” the authors should use terms such as “stimulatory” or “beneficial” for positive effects of low levels of radiation (For a review see Mortazavi et al., 2014^4^).

Furthermore, all of the references provided by the authors as the evidence for induction of adaptive response are about the effects of ionizing radiation while the induction of adaptive response in animals/cells exposed to radiofrequency radiation have been clearly studied by other researchers and our team.^3^, ^5^ I hope that these comments will be useful in better understanding the role of RF radiation emitted by cell phones on the occurrence of Tinnitus.

## Conflicts of interest

The authors declare no conflicts of interest.
